# Comparison of the efficacy of once- and twice-daily colchicine dosage in pediatric patients with familial Mediterranean fever – a randomized controlled noninferiority trial

**DOI:** 10.1186/s13075-016-0980-7

**Published:** 2016-04-07

**Authors:** Adem Polat, Cengizhan Acikel, Betul Sozeri, Ismail Dursun, Ozgur Kasapcopur, Nesrin Gulez, Dogan Simsek, Mehmet Saldir, Ipek Dokurel, Hakan Poyrazoglu, Sevcan Bakkaloglu, Ali Delibas, Zelal Ekinci, Nuray A. Ayaz, Yasar Kandur, Harun Peru, Yasemin G. Kurt, Safiye R. Polat, Erbil Unsal, Balahan Makay, Faysal Gok, Seza Ozen, Erkan Demirkaya, for the FMF Arthritis Vasculitis and Orphan Disease Research in Pediatric Rheumatology (FAVOR)

**Affiliations:** Gulhane Military Medical Academy, Pediatric Rheumatology Unit, Ankara, Turkey; Gulhane Military Medical Academy, Department of Biostatistics, Ankara, Turkey; Gulhane Military Medical Academy, Institute of Health Sciences, FMF Arthritis Vasculitis and Orphan Disease Research Center (FAVOR), Ankara, Turkey; Ege University, Pediatric Rheumatology Unit, Izmir, Turkey; Erciyes University, Pediatric Rheumatology Unit, Kayseri, Turkey; İstanbul University Cerrahpasa Medical Faculty, Pediatric Rheumatology Unit, Istanbul, Turkey; Dr. Behçet Uz Children’s Training and Research Hospital, Pediatric Allergy and Immunology Department, İzmir, Turkey; Gazi University Medical Faculty, Pediatric Rheumatology Unit, Ankara, Turkey; Mersin University Medical Faculty, Pediatric Rheumatology Unit, Mersin, Turkey; Kocaeli University Medical Faculty, Pediatric Rheumatology Unit, Kocaeli, Turkey; İstanbul Kanuni Sultan Süleyman Educational and Research Hospital, Pediatric Rheumatology Unit, Istanbul, Turkey; Selcuk University Medical Faculty, Pediatric Rheumatology Unit, Konya, Turkey; Gulhane Military Medical Academy, Department of Biochemistry, Ankara, Turkey; Dokuz Eylül University, Pediatric Rheumatology Unit, İzmir, Turkey; Hacettepe University Medical Faculty, Pediatric Rheumatology Unit, Ankara, Turkey

**Keywords:** Familial Mediterranean fever, Pediatric, Colchicine, Dosage schema

## Abstract

**Background:**

In this study, we examined the efficacy and safety of a once-daily dosage schema of colchicine compared with a twice-daily dosage schema in pediatric patients with familial Mediterranean fever (FMF).

**Methods:**

In this 24-week, multicenter, randomized controlled noninferiority trial, pediatric patients newly diagnosed with FMF carrying a homozygous or compound heterozygous mutation and not receiving any treatment were included. Patients were randomly assigned using a block randomization method to receive treatment with a once- or twice-daily dosage. Clinical and laboratory characteristics and medication side effects were recorded and compared between groups. The study was carried out in compliance with Good Clinical Practice and the Consolidated Standards for Reporting of Trials (CONSORT) statement.

**Results:**

A total of 92 patients were selected, and 79 patients completed the study. There were 42 patients in the once-daily dosage group and 37 in the twice-daily dosage group. The results indicated that the once-daily dosage was not inferior to the twice-daily dosage regarding decrease in attack frequency and duration as well as improvement in clinical findings and Mor severity scores. Alterations in laboratory findings indicating inflammation, such as erythrocyte sedimentation rate, C-reactive protein, and serum amyloid A, were similar in both groups. The rates of drug side effects were similar between the once- and twice-daily dosage groups, implying comparable safety of colchicine, with the exception of diarrhea, which was slightly higher in the once-daily dosage group.

**Conclusions:**

Using colchicine with either a once- or twice-daily dosage provides similar clinical and laboratory improvements. Considering both efficacy and safety, colchicine can be prescribed with a once-daily dosage.

**Trial Registration ID:**

ClinicalTrials.gov identifier NCT02602028. Registered 5 November 2015.

## Background

Familial Mediterranean fever (FMF) is the most frequent autoinflammatory disease. Attacks are characterized by periodic fever lasting 12–72 h and serositis, mostly manifesting as abdominal pain and resolving spontaneously [1, 2]. The disease is frequently observed, especially in the Mediterranean region among Sephardic Jews, Armenians, Turks, and Arabs [3]. The most important complication of FMF is amyloidosis. Colchicine has been shown to be the standard treatment of choice. Colchicine also has been found to be effective in decreasing the prevalence of amyloidosis [4].

Colchicine shows its main effect in mononuclear cells, where it acts as a microcompartment and keeps colchicine inside. This provides an intracellular half-life of about 35–40 h, which is longer than colchicine’s plasma half-life of about 10–20 h [5, 6]. In addition, it was previously reported that there was no correlation between plasma and intracellular colchicine concentrations, which was also shown to be unrelated to daily dosage. Higher plasma concentrations of colchicine can raise the intracellular concentration to some extent, and the concentration in mononuclear cells reaches a plateau [7, 8]. Furthermore, a pharmacodynamics study showed the effect of colchicine to be related to colchicine concentration rather than to plasma concentration in leukocytes [9].

Daily colchicine use has been shown to reduce the frequency, severity, and duration of attacks [10, 11]. Approximately 10–15 % of patients are resistant to colchicine treatment. It has been proposed that these patients are in fact noncompliers rather than nonresponsive to treatment [12]. Recently, European League Against Rheumatism recommendations for the management of FMF were published. According to the recommendations, colchicine is given in single or divided doses, depending on the tolerance and compliance of the patient [13]. It is also known that giving a drug in multiple doses reduces the patient’s adjustment to treatment [14]. For that reason, regular colchicine use is important to protect against the most feared complication associated with its use—amyloidosis—and reduce mortality. Colchicine can be used up to a dose of 2 mg/day for pediatric patients with FMF. This study was performed to investigate once- and twice-daily dosage regimens of colchicine in patients requiring a total dose of 1 mg/day [15].

Given that colchicine’s half-life is higher in mononuclear cells and that the intracellular concentration of colchicine reaches a plateau regardless of the repeated doses, theoretically there would not be any pharmacokinetic difference between once- and twice-daily dosage schemas. We therefore planned to carry out a randomized controlled trial to examine the effectiveness of colchicine treatment in control of disease symptoms, amelioration of laboratory findings, and any drug side effects (i.e., safety).

## Methods

### Study design

This study was conducted by members of the FMF Arthritis Vasculitis and Orphan Disease Research in Pediatric Rheumatology (FAVOR; www.favor.org.tr) study group at ten centers in Turkey. It was a multicenter randomized controlled trial of two parallel groups being followed in pediatric rheumatology outpatient clinics. The randomization was done at the baseline visit, and patients were assessed in two more visits 3 months apart. The study was carried out in compliance with Good Clinical Practice and the Consolidated Standards for Reporting of Trials (CONSORT) statement [16]. Ethical approval to conduct the study was obtained from Gulhane Military Medical Academy. Patients were enrolled between October 2011 and April 2013.

### Participants

#### Inclusion criteria

Pediatric patients who were newly diagnosed with FMF according to the criteria of Yalçınkaya et al. [17] or Livneh et al. [18] and who were confirmed by genetic analysis to have compound heterozygous or homozygous mutations were enrolled. Eligible patients between the ages of 5 and 16 years who weighed 15–30 kg and had not received any treatment were included. All patients included were asked by their physicians whether they had been proven and recorded to have had at least one FMF attack before enrollment in the study. An FMF attack was defined as presenting with clinical findings of fever above 38 °C lasting less than 72 h and accompanied by abdominal pain, chest pain, erysipelas such as erythema and/or swelling in the joints, and laboratory findings demonstrating an acute phase response.

#### Exclusion criteria

We excluded patients with a major congenital malformation; a risk of pregnancy; chronic diseases such as organ transplant, hepatic disorder, chronic kidney disease, amyloid A amyloidosis, and thyroid disease; or rheumatologic disorders other than FMF.

### Baseline assessment

A signed informed consent form was obtained from the legal guardian of the patients younger than 12 years of age and from both the patient and the patient’s legal guardian for patients 12 years of age and older. At the baseline visit, the patient’s medical history and complaints about the disease were questioned, and the disease features in the 6 months before admission were recorded. A physical examination and laboratory tests were performed at each visit. In the following visits, any disease attacks or findings due to colchicine treatment since the last visit were questioned and physical examination and laboratory analysis were made.

### Interventions

#### Dosage protocol and data collection

The once-daily dosage group was given colchicine once daily at 8:00 a.m., and the twice-daily dosage group received the treatment at 8:00 a.m. and 8:00 p.m. Patients were asked to visit the hospital once in 3 months for control and were evaluated in a total of three clinic visits. At the baseline visit, the patient’s medical history and complaints about the disease were questioned, and a physical examination and laboratory tests were performed. In the following visits, any attack or findings due to colchicine since the last visit were investigated in addition to the baseline visit. At the baseline and last visits, disease severity was assessed using the Mor scoring system as modified for pediatric patients [19]. The assessment of safety and tolerability of the drug was done in clinical visits every 12 weeks via physical examinations and laboratory tests. Patients were asked to note any adverse events after starting colchicine treatment and were questioned about adverse events at all visits. A physician from each center was chosen to be responsible for data collection. After each visit, the data were entered into a web-based registry system at the www.favor.org.tr website. To ensure accurate, complete, and reliable data, the following procedures were followed: (1) data collection, encoding, and storage were provided for the centers; (2) a training session was held to provide instruction on the protocol; (3) periodic meetings were held with study coordinators; (4) the principal investigator stayed in contact with the study coordinators by mail, telephone, and/or fax; and (5) a data manager reviewed and evaluated the data.

#### Colchicine dosage

The required colchicine dosage was calculated as a total of 1 mg daily according to an internationally accepted advisory published by Kallinich et al. [20]. According to the advisory, patients between 5 and 10 years of age should receive colchicine 1 mg/day, which was the age range of our patients in both groups. All patients were prescribed 0.5-mg colchicine tablets. Patients in the once-daily dosage group were given two colchicine tablets at 8:00 a.m. The twice-daily dosage group received one 0.5-mg colchicine tablet at 8:00 a.m. and one 0.5-mg colchicine tablet at 8:00 p.m. Thus, the total dosage was given in either one dose or two divided doses.

### Laboratory analysis

All samples were collected in attack-free periods. At each visit, complete blood count, urinalysis, erythrocyte sedimentation rate (ESR), C-reactive protein (CRP), blood urea nitrogen, creatinine, alanine aminotransferase (ALT), and aspartate aminotransferase (AST) tests were performed. To assess the inflammatory response, white blood cell (WBC) count, platelet (PLT) count, ESR, serum CRP, and serum amyloid A (SAA) were measured.

SAA was measured by enzyme-linked immunosorbent assay using commercial kits (CUSABIO, Wuhan, China) in accordance with the manufacturer’s instructions. The minimum detectable concentration for SAA was 39 ng/ml. Measurements were carried out using a Synergy HT microtiter plate reader (BioTek Instruments Inc., Winooski, VT, USA). All the samples were measured in duplicate.

Samples were studied in the laboratory of the department of biochemistry and FAVOR inflammation and research laboratories, Gulhane Military Medical Academy. Alterations in clinical manifestations, laboratory findings, disease severity scores, and findings due to colchicine side effects at each visit in the once- and twice-daily dosage groups were compared.

### Outcomes

The primary objectives of this study were to compare the effectiveness of once- and twice-daily colchicine dosage regimens regarding control of disease symptoms, reducing disease severity assessed using the modified Mor scoring system [21], and laboratory findings indicative of inflammation, such as ESR, CRP, and SAA. The secondary objective of the study was to evaluate the safety and tolerability of the drug and research whether once- and twice-daily dosage schemas are clinically similar with regard to side effects.

### Sample size estimation

A sample size for each arm was calculated as 43 patients per group with an α of 0.05 and 0.8 power. The noninferiority for the once-daily dosage schema was defined as a decrease in Mor score and a statistically significant decrease in the number and duration of attacks. The expected decreases in Mor scores were calculated to be 60 % for the twice-daily dosage group and 70 % for the once-daily dosage group. The noninferiority limit was assumed to be 15 %. The calculated sample sizes were smaller than the one for Mor score for decreasing the level of number and duration of attacks. For the safety analysis, clinical and laboratory adverse events were recorded for both once- and twice-daily dosage schemas. Researchers recorded and evaluated the findings after treatment was started and judged whether they were due to the study drug.

### Randomization

Block randomization into two groups was used to select groups to ensure accumulative balance of participants between arms. Computer-based block randomization algorithm was used with a block size of 2 and each patient was assigned to a treatment group with an equal chance of allocalization.

### Statistical analysis

Data analysis was performed according to the intention-to-treat principle. The clinical and laboratory findings before and after colchicine treatments were compared in the once- and twice-daily dosage groups. The changes in these parameters between visits and between groups were compared. Frequencies and percentages were used as descriptive statistics for categorical variables. To describe scale variables, mean ± SD and median (min-max) were calculated. Differences between groups were assessed by using Student’s *t* test for variables with normal distribution and the Mann-Whitney *U* test for other variables. To compare continuous variables for three visits, analysis of variance for repeated measures was used. χ^2^ tests were applied to compare discrete variables. *p* values less than 0.05 were considered significant. IBM SPSS Statistics for Windows 21.0 software (IBM, Armonk, NY, USA) was used for analysis of the data [22].

## Results

**Fig. 1:**
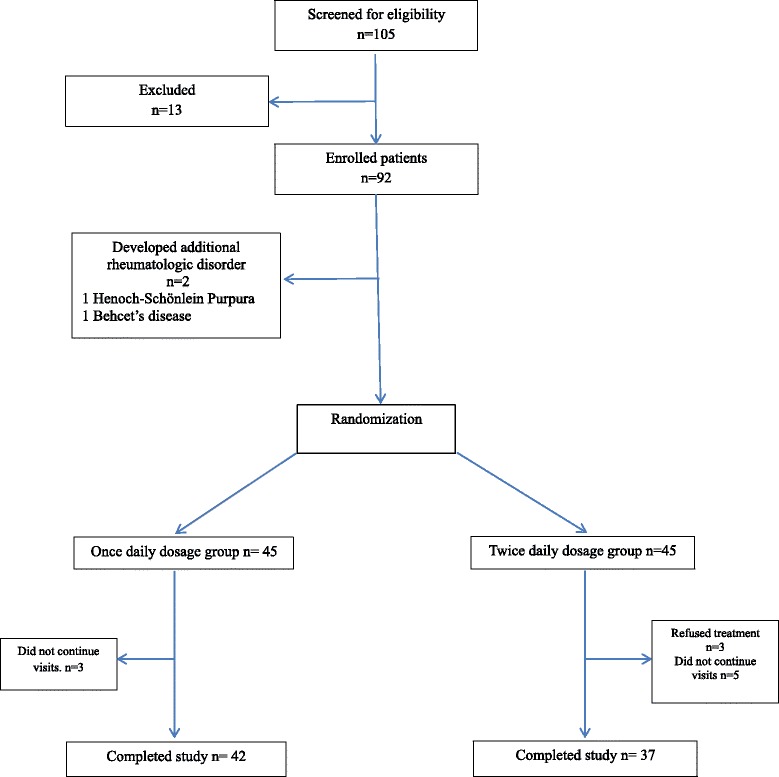
Flowchart of the patients through the study. A total of 92 patients were eligible for the study. Two of the patients were excluded due to having additional rheumatologic diseases. After randomization, three patients in the once-daily dosage group and five patients in the twice-daily dosage group were lost to follow-up. In the twice-daily dosage group, three patients refused treatment

A total of 105 patients were screened for eligibility, and 92 were selected to be included in the study. Two of the patients were excluded due to developing additional rheumatologic diseases (one with Henoch-Schönlein purpura and one with Behçet’s disease). After randomization, there were 45 patients in each group. In the once-daily dosage group, three patients did not continue their visits; in the twice-daily dosage group, three patients refused the treatment and five patients did not continue their visits. Ultimately, there were 42 patients in the once-daily dosage group and 37 in the twice-daily dosage group who completed the study (Fig. 1). The female-to-male ratios were 21:21 in the once-daily dosage group and 18:19 in the twice-daily dosage group. The participants’ mean ages were 7.9 (±1.96) years in the once-daily dosage group and 7.78 (±2.06) years in the twice-daily dosage group. In the once- and twice-daily dosage groups, the mean ages of disease onset were 5.14 (±2.90) years and 5.05 (±3.04) years, respectively, and the mean ages at the time of diagnosis were 7.54 (±2.50) years and 7.51 (±2.66) years, respectively (Table 1). The frequencies of the mutations were as follows: in the once-daily dosage group, M694V (60 %), M680I (13 %), and V726A (9 %); in the twice-daily dosage group, M694V (55 %), M680I (15 %), and V726A (10 %). The most frequent clinical symptom at the baseline visit was abdominal pain in both groups, with rates of 69 % and 64.9 % in the once- and twice-daily dosage groups, respectively. Abdominal pain was followed by fever (66.9 % in the once-daily dosage group, 54.1 % in the twice-daily dosage group), headache (40.5 % in the once-daily dosage group, 37.8 % in the twice-daily dosage group), and arthralgia (35.7 % in the once-daily dosage group, 51.4 % in the twice-daily dosage group).

**Table 1:** Demographic features of once- and twice-daily dosage groups

Characteristic	Once-daily dosage group (*n* = 42)	Twice-daily dosage group (*n* = 37)
Age,^a^ years	7.90 ± 1.96	7.78 ± 2.00
Sex, F/M	21/21	19/18
Age of disease onset,^a^ years	5.14 ± 2.90	5.05 ± 3.04
Age of disease diagnosis,^a^ years	7.54 ± 2.50	7.51 ± 2.66
Consanguinity, %	16.6	16.2
Presence of FMF in family members, %	59.5	62.2

*FMF* familial Mediterranean fever

^a^Mean ± SD

The results of the comparison of changes in clinical findings between visits before and after colchicine treatment in the two groups are presented in Table 2. The duration of attacks was lower in both groups after colchicine treatment. The median number of attacks also declined after colchicine treatment in both groups (*p* < 0.001 for both groups). After colchicine treatment, a significant decrease was observed in both groups for clinical findings frequently seen in patients with FMF, such as fever (>38 °C), abdominal pain, arthralgia (*p* ≤ 0.001 for all findings in the once-daily dosage group, *p* ≤ 0.001 for the twice-daily dosage group), arthritis (*p* < 0.001 for the once-daily dosage group, *p* = 0.003 for the twice-daily dosage group), and chest pain (*p* < 0.001 for the once-daily dosage group, *p* = 0.002 for the twice-daily dosage group). Other clinical findings manifesting during the disease course, such as malaise, confinement to bed during attacks, and headache also decreased significantly after colchicine treatment started (*p* < 0.05 for all findings).

**Table 2:** Within-group and intergroup comparison of clinical findings

	Once-daily dosage group (*n* = 42)				Twice-daily dosage group (*n* = 37)				
	Baseline	First visit	Second visit	*p* Value^a^	Baseline	First visit	Second visit	*p* Value^a^	*p* Value^b^
Features of disease									
Fever >38 °C, %	66.7	21.4	19	<0.001	54.1	22.2	16.7	0.001	0.272
Duration of attacks, h (mean ± SD)	48.00 ± 27.25	12.31 ± 25.18	8.4 ± 21.77	<0.001	44.57 ± 31.42	12.35 ± 24.08	5.6 ± 15.13	<0.001	0.711
Number of attacks^c^	3 (3–10)^d^	0 (0–1)	0 (0–1)	<0.001	3 (4–10)^d^	0 (0–1)	0 (0–0)	<0.001	0.521
Erysipelas-like erythema, %	7.1	0	4.8	0.174	5.4	0	0	0.135	0.383
Skeletal system manifestations									
Arthralgia, %	35.7	21.4	4.8	0.001	51.4	24.3	8.1	<0.001	0.376
Arthritis, %	26.2	2.4	2.4	<0.001	24.3	8.1	0	0.003	0.948
Serosal involvement									
Abdominal pain, %	69	23.8	21.4	<0.001	64.9	24.3	21.6	<0.001	0.840
Chest pain, %	31	4.8	4.8	<0.001	18.9	2.7	2.7	0.002	0.142
Systemic manifestations									
Malaise, %	64.3	26.2	14.3	<0.001	51.4	24.3	21.6	0.002	0.726
Headache, %	40.5	23.8	14.3	0.002	37.8	16.2	10.8	0.002	0.553
Confinement to bed during attacks, %	42.9	4.8	0	<0.001	37.8	0	2.7	<0.001	0.505

^a^Median (min-max)

^b^ *p* value for within-group comparison of manifestations before and after colchicine treatment

^c^ *p* value for intergroup comparison of manifestations

^d^Median number of attacks in the 6 months before admission

When the changes in consecutive visits were compared, decreases in the duration of attacks (*p* = 0.794) and the number of attacks (*p* = 0.446) were similar in both groups. There were no differences between the once- and twice-daily dosage groups when compared regarding changes in the following clinical findings: fever (*p* = 0.365), abdominal pain (*p* = 0.623), arthralgia (*p* = 0.218), arthritis (*p* = 0.900), and chest pain (*p* = 0.253) in consecutive visits. Amelioration of the symptoms malaise (*p* = 0.731), confinement to bed during attacks (*p* = 0.586), and headache (*p* = 0.530) in consecutive clinical visits were also similar between the once- and twice-daily dosage groups. The results of these analyses are presented in Table 2.

Disease severity was assessed using the Mor scoring system. The mean Mor score of the baseline visit was 3.48 ± 1.13 in the once-daily dosage group, and it was 3.27 ± 1.07 and significantly decreased after the treatment was started in both the once-daily (*p* = 0.001) and twice-daily (*p* = 0.001) dosage groups. The comparison of changes in mean Mor scores in consecutive visits between the two groups did not reveal any significant difference (*p* = 0.555) (Table 3).

**Table 3:** Comparison of groups according to modified Mor scoring system

	Once-daily dosage group (*n* = 42)		Twice-daily dosage group (*n* = 37)	
	Mean	SD	Mean	SD
Baseline	3.48	1.13	3.27	1.07
First visit	2.88	0.89	2.78	0.95
Second visit	2.81	0.83	2.76	0.93
*p* Value^a^	<0.001^b,c,d^		<0.001^b,c,d^	
*p* Value^e^	0.555			

^a^Analysis of variance (ANOVA) for repeated measures intragroup analysis

^b^Statistically significant difference between baseline and first visit

^c^Statistically significant difference between baseline and second visit

^d^Statistically significant difference between first and second visits

^e^ANOVA for repeated measures between groups analysis

ESR levels (*p* < 0.001 for the once-daily dosage group, *p* = 0.042 for the twice-daily dosage group) and CRP (*p* = 0.010 for the once-daily dosage group, *p* = 0.005 for the twice-daily dosage group) significantly decreased after colchicine treatment, while WBC and PLT counts did not reveal any significant changes. SAA levels were assessed for inflammation as well. There was a significant decrease in SAA levels in the once-daily dosage group (*p* = 0.004) and the twice-daily dosage group (*p* = 0.022). Levels of decreasing of SAA were similar in both groups after treatment (*p* = 0.761).

### Safety

After colchicine treatment started, side effects due to treatment were observed in both groups. Anorexia was significantly more frequent in both the once-daily (*p* = 0.006) and twice-daily (*p* = 0.018) dosage groups after treatment. Furthermore, the increase in anorexia at other visits was not significantly different between the groups (*p* = 0.545). Diarrhea was significantly higher in visits after treatment (19 % at the first visit, 2.4 % at the second visit) in the once-daily dosage group (*p* = 0.001), but it was not significantly higher in the twice-daily dosage group (*p* = 0.074) (10.8 % at the first visit, 2.7 % at the second visit). However, no difference was observed between the groups regarding changes in the number of patients with diarrhea before and after treatment (*p* = 0.403) (Table 4).

**Table 4:** Comparison of clinical and laboratory side effects of colchicine in subsequent visits

	Once-daily dosage group (*n* = 42)			Twice-daily dosage group (*n* = 37)			
	Baseline	First visit	Second visit	Baseline	First visit	Second visit	*p* Value^a^
Anorexia, %	0	19	16.7	0	10.8	16.2	0.545
Nausea, %	0	2.4	2.4	0	5.4	5.4	0.392
Diarrhea, %	0	19	2.4	0	10.8	2.7	0.403
Abdominal pain, %	0	9.5	11.9	0	10.8	8.1	0.800
Vomiting, %	0	4.8	0	0	8.1	5.4	0.290
Elevated ALT, %	2.4	9.5	7.1	2.7	10.8	2.7	0.838
Elevated AST, %	4.8	14.3	11.9	5.4	19.4	14.3	0.573

*ALT* alanine aminotransferase, *AST* aspartate aminotransferase

^a^ *p* value for the intergroup comparison of drug side effects

Transaminase levels were high in both the once- and twice-daily dosage groups at the second visit, after colchicine treatment was started, although in a small number of patients in both groups. High ALT levels were observed at the first visit in 9.5 % in the once-daily dosage group and 10.8 % in the twice-daily dosage group. High AST levels were observed at the first visit in 14.3 % in the once-daily dosage group and in 19.4 % in the twice-daily dosage group. At the third visit, both ALT (7.1 % in the once-daily dosage group and 2.7 % in the twice-daily dosage group) and AST (11.9 % in the once-daily dosage group and 14.3 % in the twice-daily dosage group) had returned to normal ranges in both groups. No significant difference between the once- and twice-daily dosage groups was observed for changes between visits in ALT levels (*p* = 0.838) or AST levels (*p* = 0.573) (Table 5). There was not any difference between groups regarding complications of FMF.

**Table 5:** Comparison of laboratory findings of acute phase reaction in three visits

	Once-daily dosage group (*n* = 42)			Twice-daily dosage group (*n* = 37)			
	Baseline	First visit	Second visit	Baseline	First visit	Second visit	*p* Value^a^
Leukocytosis, %	11.9	4.8	4.8	8.1	2.7	5.4	0.591
Thrombocytosis, %	4.8	2.4	4.8	5.4	2.7	10.8	0.329
High ESR, %	55.9	12.5	4.2	56.7	22.2	0	0.988
Elevated CRP, %	40.5	21.4	14.3	37.8	13.5	10.8	0.435
SAA,^b^ mg/L	4.86 ± 3.73		3.28 ± 3.40	4.70 ± 3.57		3.28 ± 3.46	0.449

*CRP* C-reactive protein, *ESR* erythrocyte sedimentation rate, *SAA* serum amyloid A

^a^ *p* value for intergroup comparison of laboratory results

^b^Mean ± SD

## Discussion

In our present randomized controlled noninferiority study, we aimed to compare once- and twice-daily dosages of colchicine in pediatric patients with FMF, with a focus on improvements in clinical and laboratory findings and drug side effects. Our results indicate that the groups were similar in these parameters and that disease severity scores declined with a similar tendency in both groups, implying clinical amelioration.

Limitations of our study can be stated as follows: The sample size was less than estimated in both groups; the duration of follow-up for safety assessment was 24 weeks, which might not be enough to evaluate the safety of the drug; and there is a lack of an outcome assessment for this disease.

The literature contains only 4 randomized, placebo-controlled clinical trials comprising a total of 57 patients with FMF treated with colchicine [11, 23–25]. Colchicine is the main choice of treatment for patients with FMF. It has been suggested to be prescribed for this disorder in a twice-daily dosage according to the aforementioned studies. This is a disadvantage for adjustments to a treatment that is highly effective in controlling attacks and severe complications of the disease when used properly, especially in adolescents, because taking a medicine in multiple doses brings with it the problem of forgetting to take doses.

The most frequent clinical findings were abdominal pain and fever in both groups and other clinical manifestations of our patients. Chest pain, myalgia, and arthritis were frequently seen in our patients with FMF. Within-group comparisons of these clinical findings revealed that colchicine provided reductions in the frequency of attacks, the duration of attacks, and the frequency of the clinical findings observed. Intergroup comparisons of improvements in these findings were similar. This indicated that colchicine was effective in controlling disease symptoms when used in a once-daily dosage as well as in a twice-daily dosage. Another noteworthy clinical finding was with regard to headache, which is not generally mentioned in many studies. Improvement in headache in patients in both groups in visits after colchicine treatment was started may suggest the contribution of colchicine to decreasing inflammation in the meninx as well as other serosal membranes.

To obtain objective data about the response to treatment, we used the severity assessment score developed by Mor et al. and modified by Ozen et al. as an outcome index [19, 26]. Both groups had a significant decrease in Mor severity scores after treatment, and no difference was detected between the groups regarding the level of decrease. These results suggest that a once-daily dosage of colchicine is not inferior to a twice-daily dosage in controlling disease severity in patients with FMF. In addition, when we compared the number of attacks and the durations of attacks with fever, we found a significant decrease in these clinical features compared with the 6-month period before the treatment was initiated. Although we accepted the Mor scoring system as a noninferiority outcome index, it is not validated in pediatric patients and lacks frequency and duration of attacks as severity parameters. In fact, these parameters are of importance in the assessment of the efficacy of FMF treatment, and this was stated in the recently developed criteria for assessment of FMF severity [16].

CRP, ESR, WBC count, and SAA were evaluated. SAA protein has been shown to increase with other acute phase reactants as a response to inflammation and is referred to as a parameter for follow-up of inflammation [21, 27]. After colchicine treatment, CRP, ESR, and WBC levels returned to normal levels, which indicated decreasing inflammation with the treatment. High SAA levels were detected at admission to the clinic, and these levels decreased in both groups after treatment, regardless of the dosage schema. These results suggest that colchicine was effective in reducing inflammation in pediatric patients with FMF when applied in either a once- or twice-daily dosage.

Another parameter taken into consideration in comparing the once- and twice-daily dosage schemas was the safety of the drug. Colchicine inhibits intracellular microtubular formation and thus inhibits mitosis. Most side effects are due to this inhibition, and they are seen in cells with a high proliferation rate. Gastrointestinal system side effects are the most prevalent, especially abdominal pain, diarrhea, nausea, and vomiting. In our study groups, anorexia, nausea, diarrhea, and abdominal pain were observed after colchicine treatment. This was consistent with the literature [20]. Anorexia was the prominent manifestation in both groups after treatment. Nausea and abdominal pain complaints did not demonstrate any significant difference before and after treatment initiated in both groups. Diarrhea was significantly high in the once-daily dosage group after treatment, but the increase in the twice-daily dosage group did not reach statistical significance. However, no patient dropped out of the study due to chronic diarrhea, as the diarrhea continued for a maximum of 1 week and none of the patients were hospitalized for diarrhea treatment. When we compared the rise in the number of patients with this complaint, we did not see any difference between the once- and twice-daily dosage groups. This suggested the complaint to be irrelevant to dosage schema.

The half-life of colchicine in plasma is 10–20 h, and it reaches 40 h in leukocytes, the site of its action [5, 8]. Taking this into consideration, there should not be any obstacle to the application of colchicine in a once-daily dosage. Starting from this point in this study, we aimed to compare once- and twice-daily dosage schemas of colchicine treatment regarding clinical manifestations, laboratory findings, and side effect profiles. The results obtained from this study revealed that application of once- or twice-daily dosage was not different, considering the frequency and duration of attacks and other clinical manifestations and laboratory findings such as acute phase reaction. Moreover, no difference was observed in manifestations of side effects due to colchicine.

Colchicine is the most effective drug for treatment of FMF, and it should be used lifelong. During childhood, the medication is generally provided by parents; in adulthood, generally problems with adjustment to treatment occur, especially if the drug is given in divided doses. According to the results of our present study, a once-daily dosage of colchicine was as effective as a twice-daily dosage in controlling disease manifestations and improvement in acute phase reactions. In addition, there was no difference in drug side effects with either dosing regimen.

## Conclusions

This large, multicenter series in a randomized controlled noninferiority trial gives evidence supporting an already common practice to give 1 mg of colchicine daily for the treatment of FMF. Colchicine treatment can be administrated in a single dose, and there is no difference between once- and twice-daily colchicine dosage schemas regarding control of clinical and laboratory manifestations or severity of the disease. Considering drug side effects in pediatric patients with FMF, both treatment schemas are similar, except for diarrhea being slightly higher in a once-daily dosage.

## Abbreviations

ALT: alanine aminotransferase
ANOVA: analysis of variance
AST: aspartate aminotransferase
CONSORT: Consolidated Standards of Reporting of Trials
CRP: C-reactive protein
ESR: erythrocyte sedimentation rate
FAVOR: FMF Arthritis Vasculitis and Orphan Disease Research in Pediatric Rheumatology
FMF: familial Mediterranean fever
PLT: platelet
SAA: serum amyloid A
WBC: white blood cell
